# Laparoscopic Repair of Catheter-Induced Intra-Peritoneal Bladder Perforation

**DOI:** 10.7759/cureus.20096

**Published:** 2021-12-02

**Authors:** Hani Sayedin, Soumendra Datta, Stephen Keoghane

**Affiliations:** 1 Urology, Warrington and Halton Teaching Hospitals NHS Foundation Trust, Warrington, GBR; 2 Urology, Colchester University Hospital NHS Foundation Trust, Colchester, GBR

**Keywords:** isolated bladder injury, laparoscopic urinary bladder repair, intra-peritoneal bladder perforation, urinary catheter related complications, iatrogenic bladder injury

## Abstract

Urinary bladder is the most common urologic organ exposed to iatrogenic injury. The bladder trauma is classified into extra-peritoneal, intra-peritoneal, or combined trauma. Intra-peritoneal bladder injury is conventionally being treated with open surgical repair, mainly to explore the abdominal viscera for possible associated injuries and to insert peritoneal drain. One rare form of the iatrogenic bladder injury is catheter-related bladder injury which is very uncommon and only few cases were reported. It is mainly related to other associated medical conditions like cancer and chronic catheterization which might be causing subsequent bladder wall weakness. Therefore, it is important to collect more data about this rare type of bladder injury, particularly urethral catheterization which is one of the most common medical procedures. We present a 74-year-old male patient who developed acute kidney injury and was treated by urethral catheterization in the emergency department. The patient developed immediately severe abdominal pain. Non-contrast CT showed intra-peritoneal bladder perforation by the urethral catheter. The patient developed peritonitis and failed a trial of conservative management. Consequently, laparoscopic abdominal exploration and bladder repair was performed successfully.

## Introduction

Bladder trauma can result in extravasation of urine into the extra-peritoneal space (60%), intra-peritoneal space (30%) or both (10%) [1]. The majority of extra-peritoneal injuries can be treated conservatively with catheter drainage as long as there is no other pathology requiring surgical exploration [2]. Intra-peritoneal rupture is usually caused by penetrating trauma or iatrogenic trauma or external trauma to full bladder causing rupture of the weakest part of the bladder, usually the dome. Iatrogenic intra-peritoneal bladder trauma is mainly secondary to urological and gynaecological procedures. Intra-peritoneal bladder rupture can be associated with complications including uremia, peritonitis, abscess formation, and electrolyte disturbance. Therefore, open surgical exploration and repair is considered the standard of care [3]. In addition, laparoscopic surgical repair is proven to be beneficial in the treatment of isolated intra-peritoneal perforation. Subsequently, careful assessment with CT and cystography is crucial in patient assessment. Catheter-related bladder injury is rarely reported form of the iatrogenic bladder injury. However, it might be related to chronic urethral catheterization particularly in elderly patients, or secondary to bladder disease including diverticulum, cancer, or previous irradiation. Therefore, the presentation could be subacute or delayed [4]. To the maximum of our knowledge, this is the first case to be reported that developed bladder perforation directly on catheter insertion resulting in acute peritonitis.

## Case presentation

**Figure 1:**
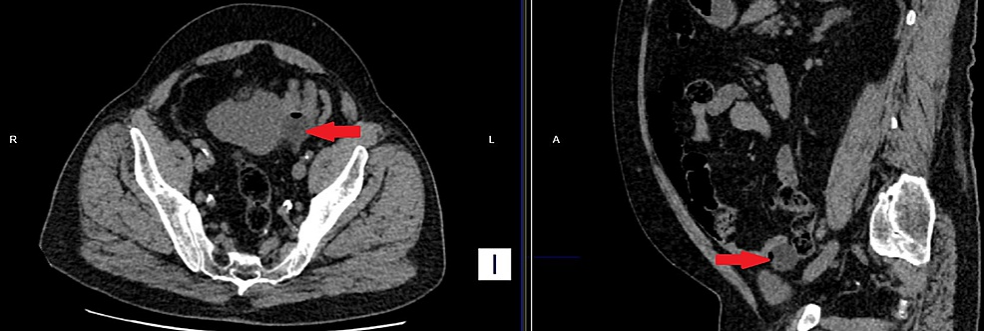
Non-contrast CT To the left, the axial cut view shows the inflated catheter balloon (red arrow) outside the bladder. To the right, the sagittal cut view shows the balloon (red arrow) out of the bladder.

**Figure 2:**
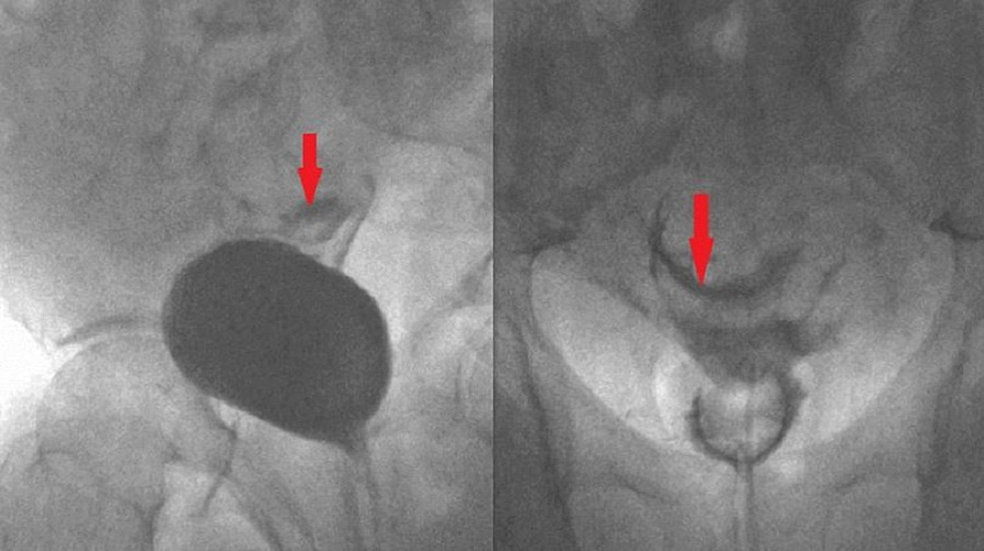
Ascending cystogram - Fluoroscopy images To the left, the oblique view of the filling phase shows contrast extravasation (red arrow). To the right, the post-micturition phase shows the contrast extravasated into the intra-peritoneal cavity (red arrow).

A 74-year-old male patient presented to the emergency department (ED) complaining of abdominal pain. The patient has a medical history of chronic obstructive pulmonary disease (COPD) and diverticulosis. In addition, he has a surgical history of colo-vesical fistula that was repaired by laparoscopic left hemi-colectomy and closure of the fistula four years back, and transurethral resection of the prostate (TURP) two years back for lower urinary tract symptoms (LUTS) management. In the ED, the patient was provisionally diagnosed with acute kidney injury (AKI), serum creatinine was 201 µmol/L and eight months before was 92 µmol/L. Therefore, a decision was taken to insert a urethral catheter. Two-way 16 Fr Foley catheter was inserted resulting in worsening abdominal pain and signs of peritonitis, pain score was 8 out of 10 on the numeric rating scale (NRS). A non-contrast CT revealed a possible intra-peritoneal bladder perforation by the catheter (Figure 1). The urethral catheter was not draining. The urethral catheter was replaced with another two-way 16 Fr. The new catheter was inserted slowly which then started to drain urine. The patient’s pain significantly improved, and pain score went down to 5 out of 10. Two hours later, a cystogram was done and demonstrated the newly inserted urethral catheter to be in the correct position but there was intra-peritoneal extravasation of contrast confirming an intra-peritoneal bladder injury (Figure 2).

The patient was re-assessed after the cystogram. He was much more comfortable and he reported marked improvement of the pain, score 3 out of 10. On examination, the abdomen became much more laxer with mild tenderness only at the lower abdomen. Besides, the patient had no fever, WBCs count and C-reactive protein (CRP) were normal. Therefore, we conducted a discussion with the patient and we agreed about a trial of conservative management with IV fluids and parenteral antibiotics.

After admission, the patient developed an increase in the abdominal pain intensity, pain score raised to 5 out of 10, and guarding started to occur. In addition, bladder spasms bouts made the pain more intense. The patient venous blood gas (VBG) showed normal lactate and there was no fever. The kidney function recovered well to the IV fluid and serum creatinine improved to 115 µmol/L. Repeated full blood count showed normal WBCs. However, the patient showed clinical signs of deteriorating peritonitis and the pain became intolerable, and the score raised back to 8 out of 10. In addition, patient-controlled anesthesia (PCA) was stopped because the patient was already on oxygen supply at a rate of 4 L/min to maintain arterial O2 saturation at 90%, considering that he already has COPD.

**Figure 3:**
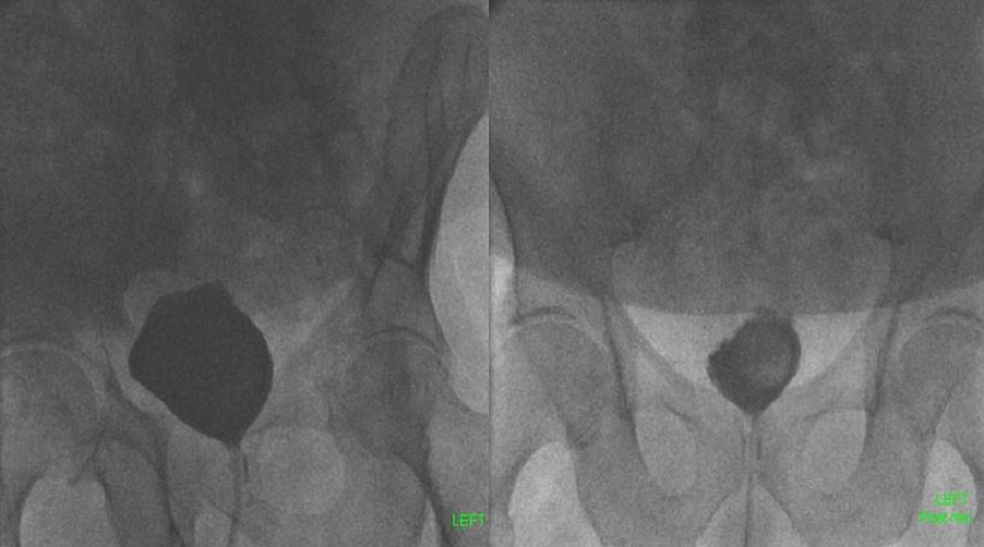
Ascending cystogram two weeks post-operative repair To the left filling phase and to the right post-micturition phase, both show no contrast extravasation.

A clinical deterioration led to operative intervention and a laparoscopic exploration was performed and the bladder was repaired using 2/0 vicryl in two layers. A peritoneal drain 14 Fr was left at the end of the procedure and was removed on the third postoperative day and the patient was discharged with a urethral catheter 16 Fr. Two weeks later a cystogram demonstrated no leak or extravasation (Figure 3), the urethral catheter was removed in the same day.

## Discussion

Catheter-induced intra-peritoneal bladder injury is a rare form of iatrogenic bladder trauma. It may be related to long-term catheterization, i.e., catheter-induced spontaneous rupture. Magee et al. reported an intra-peritoneal bladder rupture in a 76-year-old male patient with a long-term catheter [4]. The patient developed peritonitis and the diagnosis was made at surgical exploration. Zhan et al. reported another case of catheter-related intra-peritoneal bladder rupture in an 83-year-old male patient with a long-term urethral catheter who presented with haematuria and delirium [5]. The patient was successfully treated with conservative management, which included adequate bladder drainage. This report showed the importance of a CT scan to achieve the diagnosis. Ogawa et al. reported an intra-peritoneal perforation in an 86-year-old, related to a long-term indwelling catheter [6]. Contrast CT confirmed the diagnosis and in spite of an early diagnosis and surgical repair, the patient was hospitalized for 97 days due to post-operative complications. There are also reports that document long-term catheter-induced extra-peritoneal bladder rupture [7].

Jambet et al. reported a case of catheter-induced intra-peritoneal perforation in a vasculopath who developed urinary retention following a vascular procedure [8]. The authors reported that the patient pulled his catheter on two occasions due to alcohol withdrawal resulting in abdominal pain. He was explored for suspected mesenteric ischaemia; the catheter injury was detected intra-operatively.

Poola and Mohan reported a catheter-induced intra-peritoneal perforation in a 62-year-old woman with multiple co-morbidities including diabetes mellitus, hypertension, and end-stage renal disease [9]. The patient developed urinary retention and a catheter was inserted. Twenty-four hours following catheterization, the patient developed abdominal pain and sepsis. Non-contrast CT and a CT cystogram confirmed the diagnosis but despite the surgical repair, she developed post-operative complications and died.

Catheter-related bladder injury is very uncommon and occurs in elderly patients with a long-term catheter. Chronic irritation and bacteriuria might play role in bladder wall weakness facilitating the bladder injury. However, the presentation in such cases is subacute or delayed. Furthermore, the poor general condition might worsen the prognosis. Our case has a history of colo-vesical fistula repair and mostly resulted in a weak scar at the bladder wall where the catheter-related injury occurred. CT and cystography confirmed the isolated injury. However, he failed the conservative management because of the acuity of the injury. Moreover, laparoscopic bladder repair could be equivalent to open surgery with a satisfactory outcome.

## Conclusions

Catheter-related bladder injury is an uncommon complication of urethral catheterization; however, it is a very devastating complication and might be fatal, particularly if missed. If the patient developed signs of peritonitis post urethral catheterization, CT and cystography might be recommended for thorough assessment. Conservative management, if adopted, should be under close observation and surgical intervention should be undertaken without delay if needed. Furthermore, a laparoscopic repair could be performed with equivalent efficiency to open surgery. Considering the extreme common use of urethral catheter, treating physician needs to be aware of the rare possibility of catheter-related bladder perforation.
